# Transcriptomic profile comparison reveals conservation of ionocytes across multiple organs

**DOI:** 10.1038/s41598-023-30603-1

**Published:** 2023-03-02

**Authors:** Carla Pou Casellas, Cayetano Pleguezuelos-Manzano, Maarten B. Rookmaaker, Marianne C. Verhaar, Hans Clevers

**Affiliations:** 1 Oncode Institute, Hubrecht Institute, Royal Dutch Academy of Science (KNAW) and University Medical Center Utrecht (UMCU), Utrecht, The Netherlands; 2 Department of Nephrology and Hypertension, University Medical Center Utrecht (UMCU), Utrecht, The Netherlands; 3 Present Address: Roche Pharmaceutical Research and Early Development, 4058 Basel, Switzerland

**Keywords:** Systems biology, Biomarkers

## Abstract

Single-cell RNA sequencing has recently led to the identification of a flurry of rare, new cell types, such as the CFTR-high ionocytes in the airway epithelium. Ionocytes appear to be specifically responsible for fluid osmolarity and pH regulation. Similar cells exist in multiple other organs and have received various names, including *intercalated cell* in the kidney, *mitochondria-rich cell* in the inner ear, *clear cell* in the epididymis, and *ionocyte* in the salivary gland. Here, we compare the previously published transcriptomic profile of cells expressing *FOXI1*, the signature transcription factor expressed in airway ionocytes. Such *FOXI1*+ cells were found in datasets representing human and/or murine kidney, airway, epididymis, thymus, skin, inner ear, salivary gland, and prostate. This allowed us to assess the similarities between these cells and identify the core transcriptomic signature of this ionocyte ‘family’. Our results demonstrate that, across all these organs, ionocytes maintain the expression of a characteristic set of genes, including *FOXI1*, *KRT7,* and *ATP6V1B1*. We conclude that the ionocyte signature defines a class of closely related cell types across multiple mammalian organs.

## Introduction

Regulation of ion osmolality in epithelial surfaces is a key process for the correct functioning of many epithelial organs, being central to the maintenance of luminal fluid volume, mucus viscosity and regulation of pH. Different types of ion- and pH-regulating cells have been described in vertebrates. They were first reported in eel gill epithelium, and subsequently identified both in gills of fresh and seawater fish, as well as in *Xenopus* epidermis^1–3^. More recently, and despite the inconsistent terminology, ion-secreting cells have been described in several mouse and human organs using single-cell RNA sequencing (scRNA-seq) approaches. In the kidney, intercalated cells regulate urine pH in the collecting duct^4^. In the airway, ionocytes are suggested to regulate osmolarity, pH and viscosity of the airway surface liquid and mucus^5,6^. In the inner ear, mitochondria-rich cells (MRC) regulate the endolymphatic electron potential required for hearing^7^. In the epididymis, clear cell-mediated luminal acidification contributes to sperm maturation and fertility^8,9^. Furthermore, other ion-secreting populations have been recently described in the thymus, and in salivary and sweat glands^10–12^. For clarity, we hereinafter refer to all these mammalian ion-secreting cells as ionocytes.

Because of their central role to the functioning of many different organs, ionocytes have been implicated in the onset of various diseases. As a clear example, mutations in the chloride channel *CFTR,* an ionocyte marker, give rise to cystic fibrosis (CF). CF is characterized by a desiccated mucus layer in the airway, male infertility, salt imbalance in sweat, and sometimes, kidney disorders^13–16^. As another example, malfunction of other ionocyte-related genes, namely vacuolar ATPases, can cause distal renal tubular acidosis accompanied by hearing loss^17^. This highlights the functional (dis)similarities of ionocytes across different organs.

**Figure 1:**
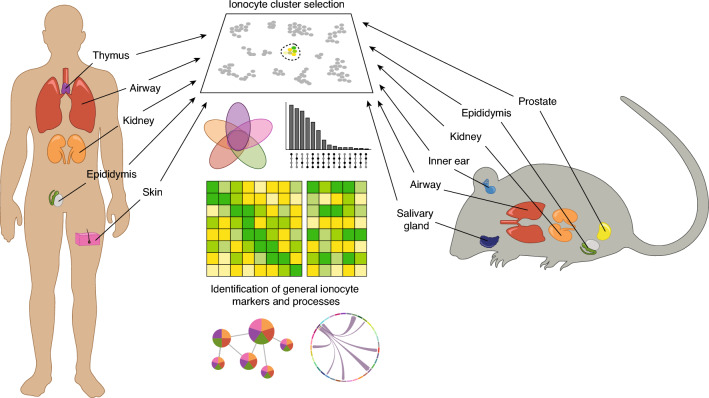
Strategy applied in this study to identify the core ionocyte gene signature in humans and mice.

Despite the large number of scRNA-seq datasets available to date, a systematic comparison of the development and function of ionocytes across different mouse and human organs has not been reported to our knowledge. In this report, we compile and compare scRNA-seq datasets from human thymus, airway, kidney, epididymis and skin, and mouse prostate, epididymis, kidney, inner ear, airway and salivary gland. This analysis allows us to define a core ionocyte gene signature and to establish differences that are specific to ionocytes between tissues. In this way, the detailed characterization of ionocytes across several human and mouse tissues contributes to a better comprehension of this cell type and provides a framework to better understand ionocyte functional dysregulation, its involvement in disease pathophysiology and its potential as diagnostic biomarker (Fig. 1).

## Methods

### Data processing and clustering

The human and murine scRNA-seq datasets used in this study can be found in Table 1. To ensure the highest data quality and comparability, we exclusively included datasets of healthy adult (or at least postnatal) subjects/animals and containing cells expressing forkhead box I1 (*FOXI1)*. In line with this, from the dataset by Hauser et al.^18^ we only used data from P30 and adult mice in our study.

**Table 1:** List of single-cell RNA sequencing datasets used in this study.

Species	Tissue of Origin	Study ID	References
Human	Kidney	GSE151302	Muto et al.^26^
	Airway	GSE102580	Plasschaert et al.^27^
		GSE166766	Ravindra et al.^28^
		syn21041850	Travaglini et al. ^29^
	Epididymis	GSE148963	Leir et al.^30^
	Thymus	GSE147520	Bautista et al.^10^
	Skin	GSE147424	He et al.^11^
Mouse	Kidney	GSE107585	Park et al.^31^
		GSE129798	Ransick et al.^32^
	Airway	GSE103354	Montoro et al.^5^
		GSE102580	Plasschaert et al.^27^
	Epididymis	GSE159713	Shi et al.^33^
		GSE145443	Rinaldi et al. ^34^
	Inner ear	GSE87293	Honda et al.^35^
	Salivary gland	GSE150327	Hauser et al.^18^
	Prostate	GSE146811	Karthaus et al.^36^

All raw data files were processed and analyzed using the Seurat package (v.4.1.0)^19^ in R (v.4.0.3). For all datasets, data filtering was performed equally, requiring a minimum of 5 cells per transcript, at least 300 transcripts per cell, and less than 5% expression of mitochondrial genes per cell. After filtering and normalization using the *NormalizeData* command, individual datasets or datasets belonging to the same organ were integrated using either Seurat’s canonical correlation analysis (CCA) or reciprocal principal component analysis (PCA) methods. Upon subsequent data scaling, PCA and Uniform Manifold Approximation and Projection (UMAP) dimensionality reductions were run to create UMAP visualization plots. Clustering was performed using the functions *FindNeighbors* and *FindClusters*, and the ionocyte cluster(s) in each organ dataset were identified based on the highest expression of *FOXI1*.

### Marker differential expression and biological process analysis

The top 25 differentially expressed genes (DEGs) per ionocyte population were generated using Seurat’s *FindMarkers* function selecting a minimum log2 fold-change of 0.25. The top 20 significantly upregulated biological processes based on these DEGs were computed using the clusterProfiler package (v.3.18.1)^20^ and rendered using the packages enrichplot (v.1.10.2)^21^ and DOSE (v.3.16.0)^22^.

### Common feature (gene intersection) analysis

Venn diagrams and gene intersection bar plots were created using the positive DEG lists per ionocyte population with the R packages VennDiagram (v.1.7.1)^23^ and UpSetR (v.1.4.0)^24^, respectively.

### Integration of all human and murine datasets and correlation analysis of human data

All individual human datasets were integrated together by reciprocal PCA, using a k.weight of 80. Afterwards, the integrated object’s data was scaled, dimensionality reductions were run, and clustering was computed. Differentially expressed markers in each cluster were calculated using a log fold-change threshold of 0.5. The same applies for the integration of all murine datasets. Correlation of average expression was subsequently performed comparing the different identities of the object (i.e., each organ-specific ionocyte subset) using the *cor* function.

### Intercellular communication analysis

Cell–cell communication analysis was performed using the R package CellChat (v.1.1.3)^25^. The same workflow was applied for each ionocyte population, which were subsetted from the main integrated dataset. The computed communications were filtered based on a minimum of 10 cells.

## Results

### Ionocyte populations can be identified in at least five different human organs based on *FOXI1* expression

**Figure 2:**
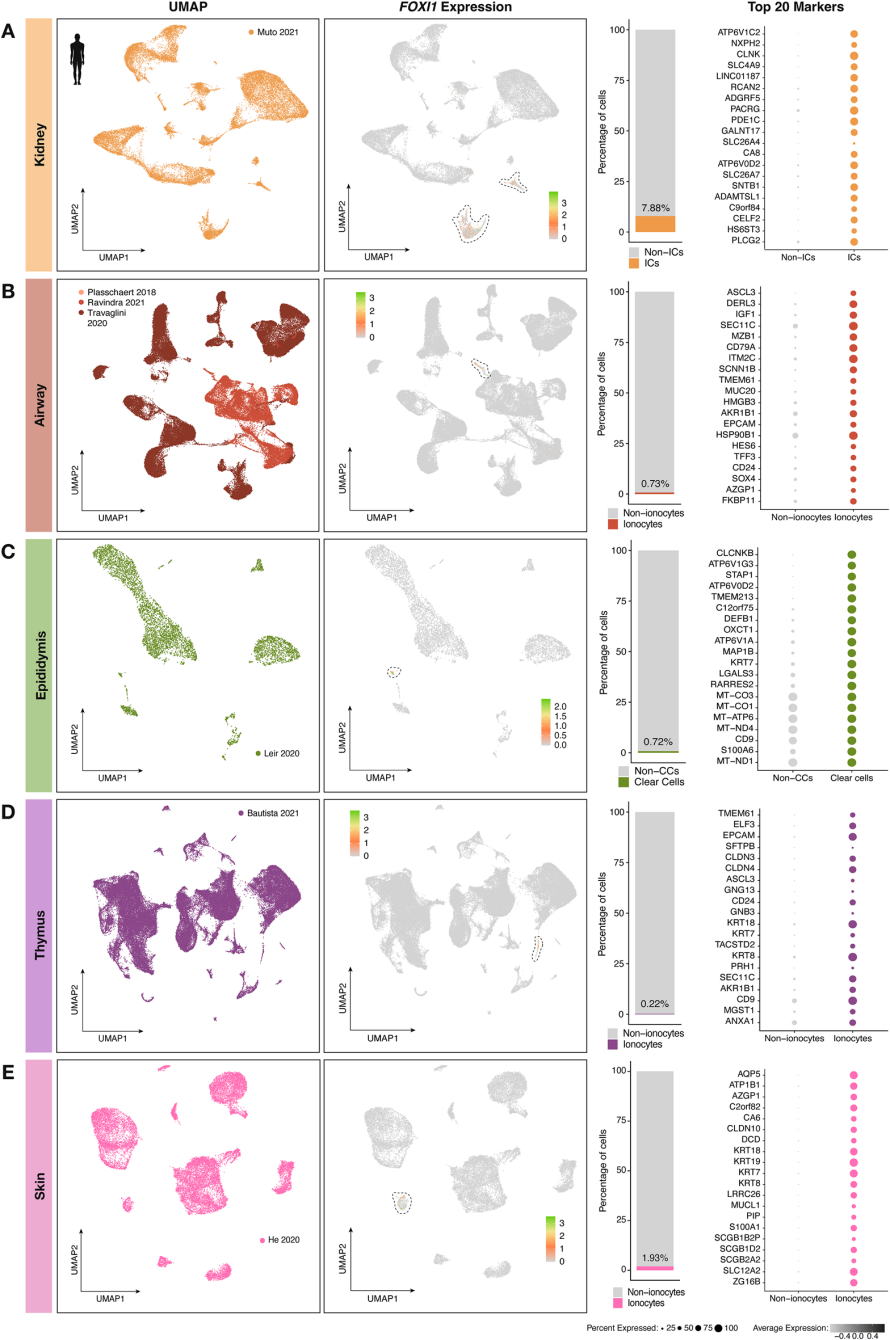
Ionocytes can be identified in several human tissues by having the highest *FOXI1* expression. Depicted are the Uniform Manifold Approximation and Projection (UMAP) plots per organ (**A**: kidney, **B**: airway, **C**: epididymis, **D**: thymus, **E**: skin), together with feature plots showing where each *FOXI1*-expressing cluster is located, and the percentage of cells found in these ionocyte clusters. Finally, the top 20 differentially expressed markers per ionocyte population can be observed.

To explore and compare the specific markers in ionocytes derived from different human tissues, we analyzed publicly available healthy tissue scRNA-seq datasets in which the presence of ionocytes was expected. To enable a precise identification of ionocytes in each dataset, we selected data files in which expression of the main ionocyte transcription factor, *FOXI1*^37^, could be measured. This resulted in the acquisition of seven datasets, profiling a total of five human organs: kidneys, airway, epididymis, thymus, and skin. The kidney dataset consisted of 26,933 cells, among which 2064 (7.88%) were identified as *FOXI1*+ intercalated cells. As expected, some of the top markers differentially expressed in these cells were the vacuolar ATPase subunit C2 (*ATP6V1C2*), the bicarbonate transporter *SLC4A9*, and the adhesion G protein-coupled receptor F5 (*ADGRF5*) (Fig. 2A), which are all well-known markers for this cell type.

Given that airway ionocytes have been so scarcely sequenced, we performed our analyses using three datasets in order to obtain a sufficiently large number of cells. The three datasets combined yielded a total of 91,226 cells, including 472 ionocytes (0.73%) defined by the expression of *FOXI1*. The top differentially expressed markers in this small population included the Achaete-Scute Family BHLH Transcription Factor 3 (*ASCL3*), insulin-like growth factor 1 (*IGF1*), and the integral membrane protein 2C (*ITM2C*) (Fig. 2B).

The employed epididymis dataset contained 5,299 cells, among which 68 (0.72%) were recognized as ionocytes, expressing high levels of markers such as the chloride voltage-gated channel Kb (*CLCNKB*), the vacuolar ATPase subunit G3 (*ATP6V1G3*), and keratin 7 (*KRT7*) (Fig. 2C). As a representation of the thymus, we found a dataset comprising 72,382 cells, and the lowest percentage of *FOXI1*+ ionocytes (0.22%, 83 cells) among the tissues studied. Thymus ionocytes differentially expressed markers including the E74-like ETS transcription factor 3 (*ELF3*), claudin 3 (*CLDN3*), and *CD24* (Fig. 2D). Finally, we used a skin dataset comprising a total of 20,385 cells and 236 (1.93%) of ionocytes. This ionocyte population expressed the highest levels of aquaporin 5 (*AQP5*), the ATPase Na^+^/K^+^ transporting subunit beta 1 (*ATP1B1*), and carbonic anhydrase 6 (*CA6*) in human skin, among other markers (Fig. 2E). The clusters in each dataset can be found in Supplementary Figs. 1A–E.

### Ionocytes display a conserved function and transcriptomic profile across different human organs

**Figure 3:**
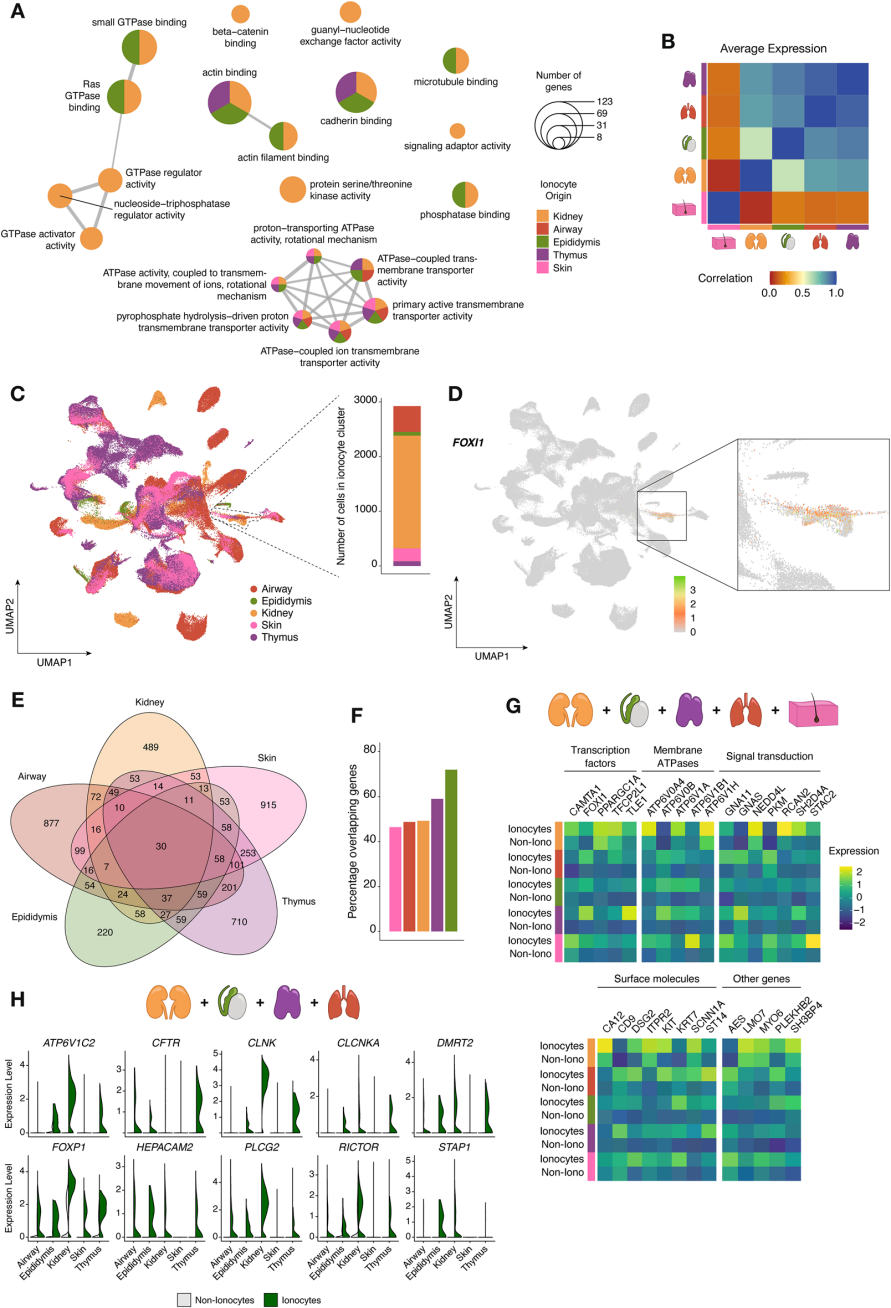
Human ionocytes derived from kidney, airway, epididymis, thymus, and skin are functionally and transcriptionally very similar. (**A**) Enrichment map depicting the most highly enriched biological processes based on the differentially expressed genes (DEGs) in each ionocyte population. (**B**) Correlation heatmap of average gene expression between ionocytes found in thymus, airway, epididymis, kidney, or skin. The correlation coefficient indicates the results of a Pearson’s correlation test. (**C**) Uniform Manifold Approximation and Projection (UMAP) plot outlining the integration of five whole-tissue human datasets. The traced cell cluster indicates the ionocyte cluster, and the bar graph shows the number of cells comprising this cluster derived from each organ. (**D**) Feature plot showing relative *FOXI1* expression in the ionocyte cluster. (**E**) Venn diagram illustrating the shared DEGs between the five ionocyte populations. (**F**) Bar graph indicating the percentage of overlapping genes between each ionocyte population and the rest. (**G**) Heatmaps depicting the relative expression levels of the 30 shared DEGs between ionocytes and non-ionocytes per tissue of origin. (**H**) Violin plots of exemplar DEGs shared between kidney, epididymis, thymus, and airway ionocytes.

Upon the preliminary identification of ionocytes in each human tissue dataset, we sought out to investigate how comparable these cells are in terms of predicted function and gene expression. Using a gene set enrichment analysis (GSEA) based on biological processes, we observed that all five ionocyte populations share similar predicted processes or functions, mainly those concerning transmembrane transport and pH regulation (Fig. 3A). As for their transcriptomic profile, we noted that the average expression of all genes was highly correlated (Pearson coefficient > 0.5) across ionocyte populations derived from thymus, airway, epididymis, and kidney. In contrast, the average gene expression in skin *FOXI* + cells correlated poorly with those of the other four organs (Fig. 3B).

To compare the human ionocyte populations in more detail, we integrated all five whole-tissue datasets and observed a distinct ionocyte cluster expressing *FOXI1,* composed of cells emerging from the five different organs (Fig. 3C, D). All clusters of the resulting integrated UMAP can be seen in Supplementary Fig. 1F, together with an overview of the markers expressed per cluster, each cluster’s identity, and the cluster composition based on tissue of origin (Supplementary Fig. 1G). After computing the list of upregulated genes in each ionocyte population, we identified 30 markers shared between all of them (Fig. 3E). Notably, each ionocyte population shared at least 40% of their differentially expressed genes with other ionocyte populations (Fig. 3F), confirming the transcriptomic similarity between these populations. The 30 markers commonly shared among all ionocytes included several transcriptional regulators (e.g., calmodulin binding transcription activator 1 (*CAMTA1)*, *FOXI1*, peroxisome proliferator-activated receptor gamma coactivator 1 alpha (*PPARGC1A*), and transcription factor CP2-like 1 (*TFCP2L1*)); membrane ATPases such as *ATP6V0A4*, *ATP6V0B*, and *ATP6V1B1*; signal transduction-related genes (e.g., guanine nucleotide binding protein alpha (*GNAS*)*,* and regulator of calcineurin 2 (*RCAN2*))*,* and surface molecules (e.g., *CA12, KIT*, and *KRT7*) (Fig. 3G). Given that *CFTR* is a well-known ionocyte marker in airways and kidney^6^ but was not found in the list of shared markers among the five populations, we explored which ionocyte populations shared a significant differential expression of this gene. We found that *CFTR* differential expression was shared between kidney, epididymis, thymus, and airway ionocytes, along with other important markers such as *CLCNKA*, doublesex and mab-3 related transcription factor 2 (*DMRT2*), forkhead box P1 (*FOXP1*), and phospholipase C gamma 2 (*PLCG2*) (Fig. 3H). The complete list of overlapping genes between each ionocyte population can be found in Supplementary File 1.

### Human ionocyte interactions and signaling are preserved among different tissues

**Figure 4:**
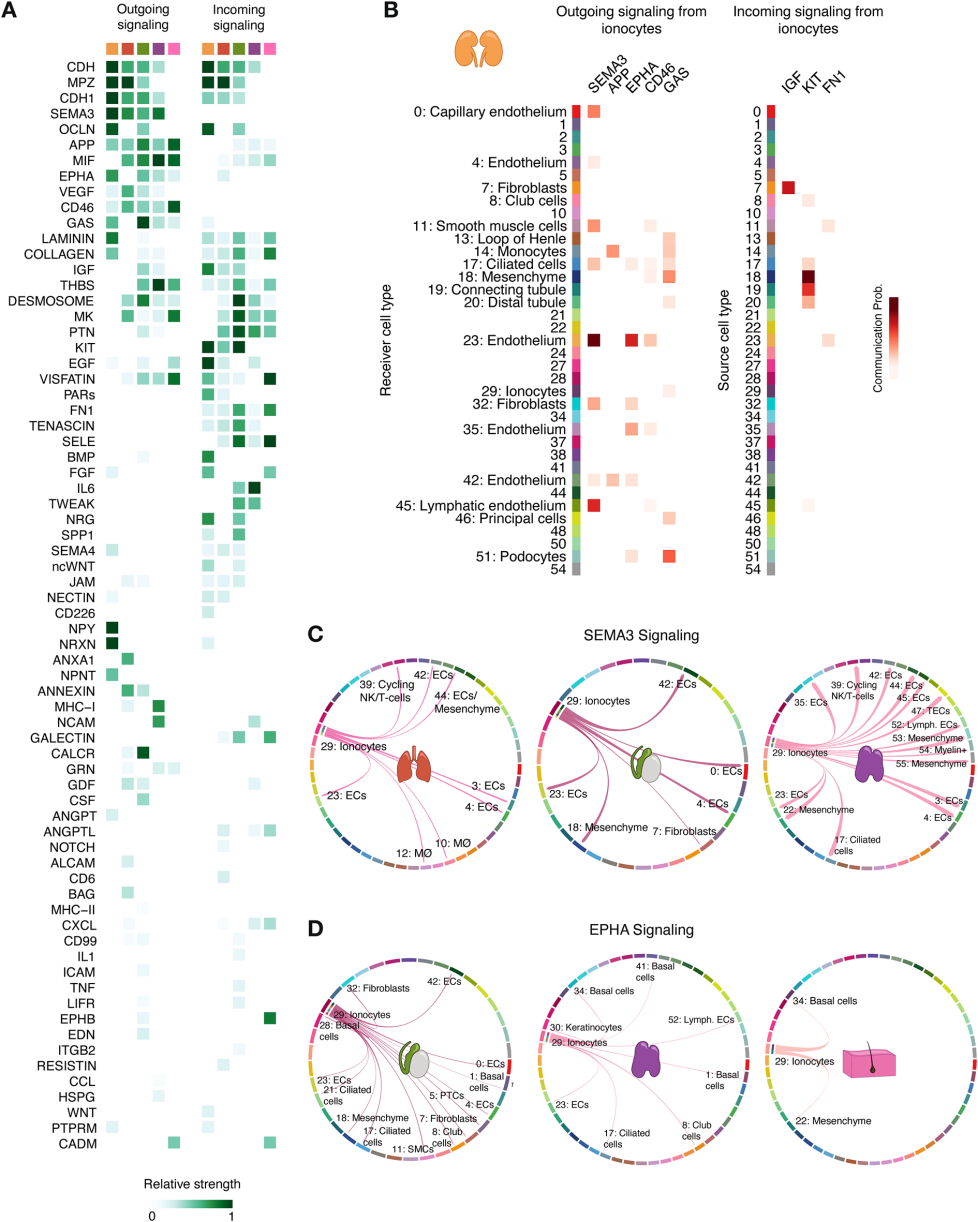
Main outgoing and incoming signaling in human ionocytes. (**A**) Heatmap showing the list of pathways identified as relevant outgoing or incoming signals in human ionocytes derived from different organs (orange: kidney, red: airway, green: epididymis, purple: thymus, pink: skin). (**B**) Heatmap depicting examples of probable communications between kidney ionocytes and other cell types through different pathways. Examples of communications between ionocyte populations from other organs and other cell types based on the signaling pathways semaphorin 3 (SEMA3) (**C**) and ephrin-A (EPHA) (**D**) can be seen as chord diagrams.

To further investigate the general ionocyte functions, we computed both the incoming and outgoing signaling pathways in ionocytes derived from the five organs in question: kidney, airway, epididymis, thymus, and skin. A summary of all involved pathways detected in each population can be observed in Fig. 4A. Signaling pathways that were both incoming and outgoing in ionocytes, mostly referring to autocrine pathways, included e-cadherin (CDH1) and myelin protein zero (MPZ) signaling. In terms of prediction of intercellular communication, we observed that the main outgoing signaling pathways from ionocytes included those capable of affecting endothelium and mesenchyme, for instance, the semaphorin 3 (SEMA3) and ephrin-A (EPHA) pathways. These findings were also reported in a recent publication where salivary glands were studied^12^. Other major pathways found to be signaled by ionocytes revolve around immune system regulation. Two clear examples are the macrophage migration inhibitory factor (MIF) and CD46 signaling. MIF is a pro-inflammatory cytokine that greatly regulates innate immunity^38^, while CD46 is thought to regulate T cell-driven inflammation^39^. The putative ability of ionocytes to modulate the immune system was exemplified in an in vivo study that showed the chemoattraction power of neutrophils by kidney intercalated cells^40^. Finally, we observed that ionocytes globally receive signals from other cells through collagen, midkine (MK), pleiotrophin (PTN), and fibronectin (FN1) signaling pathways, among others. Notably, both MK and PTN are heparin-binding growth factors^41^, indicating that heparin might be an important regulator of ionocyte function or differentiation. Among the incoming signaling pathways we also found insulin-like growth factor (IGF), which has been found to trigger salt secretion in fish^42^. Surprisingly, while several studies have reported that ionocyte development and cell state is dependent on Notch signaling^27,43^, our analysis did not find Notch to be an important signaling pathway in ionocytes (Fig. 4A).

To get an indication of the kind of receiver and source cell types that interact with ionocytes, we generated a communication probability heatmap showing examples of main pathways found in all ionocytes with a clear outgoing or incoming trend. Figure 4B depicts an illustrative heatmap showing the cellular communicators that kidney-derived ionocytes have. Figure 4C, D show examples of two of these pathways, namely SEMA3 and EPHA, in ionocyte populations derived from other organs. As observed, ionocytes derived from different human organs have similar inferred signal-receiving cell targets.

### Exploration of murine datasets reveals the presence of ionocytes in other organs

**Figure 5:**
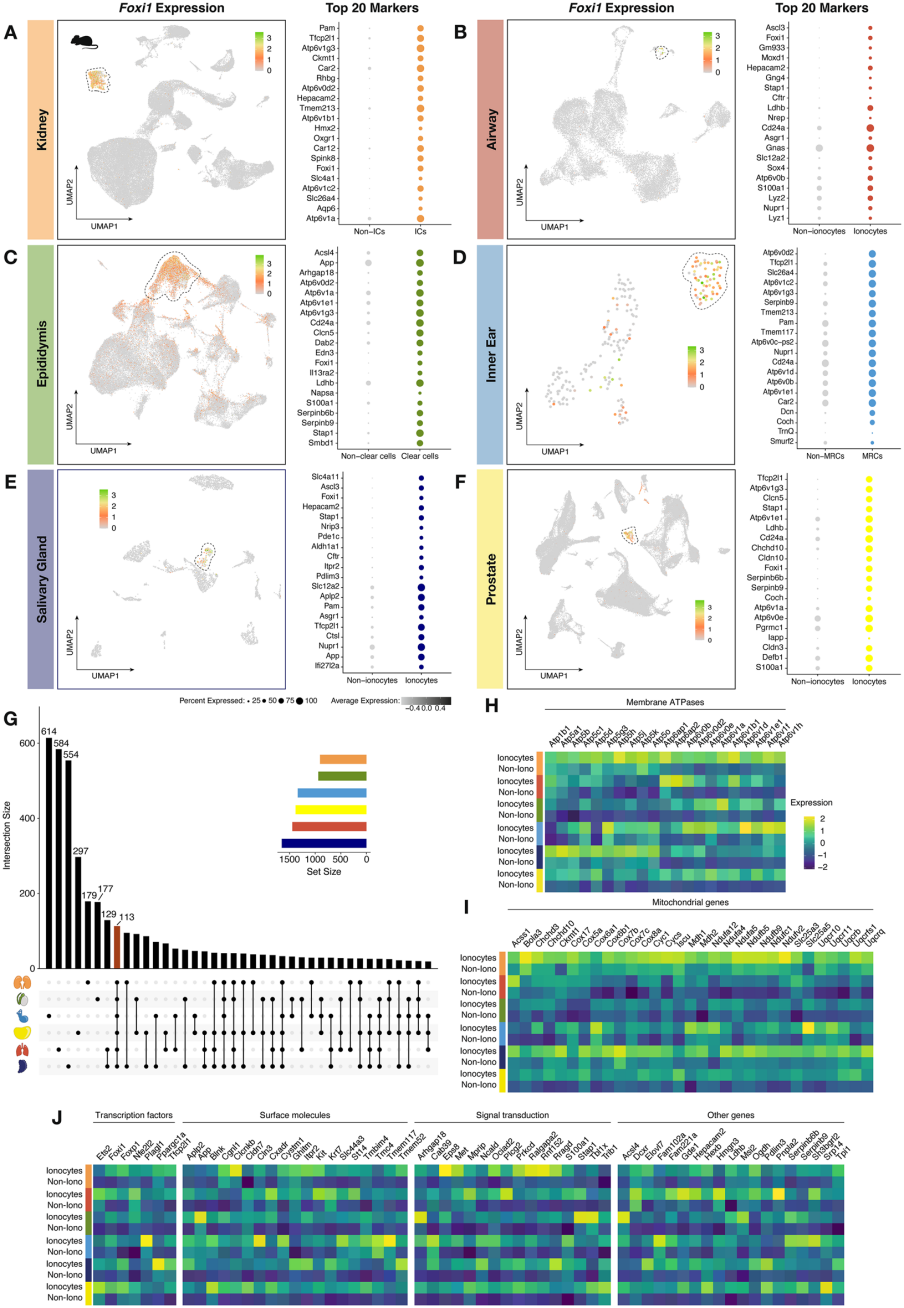
Murine ionocyte identification and comparison. Depicted are feature plots from datasets of murine tissues (**A**: kidney, **B**: airway, **C**: epididymis, **D**: inner ear, **E**: salivary gland, **F**: prostate), showing the presence of ionocyte clusters (*Foxi1*-expressing cells). Next to each feature plot, we show the top 20 differentially expressed genes (DEGs) per ionocyte population. (**G**) Bar graph revealing the intersecting DEGs between ionocyte populations. The 113 genes commonly shared in all five ionocyte populations can be observed in the heatmaps, which were divided by category (**H**, membrane ATPases; **I**, mitochondrial genes; **J**, transcription factors, surface molecules, signal transduction-related genes, and other genes).

Since human datasets were not available for all desired organs meeting our inclusion criteria, we opted for repeating the analysis using murine datasets. Thus, we could also include healthy inner ear, salivary gland, and prostate datasets. To ensure the highest data quality and representability in our study, we integrated two different datasets per organ of interest. However, for inner ear, salivary gland, and prostate we could only find one available dataset. This resulted in the inclusion of two murine kidney datasets, two airway datasets, two epididymis datasets, one inner ear, one salivary gland, and one prostate dataset. All UMAPs and computed clusters for each separate organ can be found in Supplementary Figs. 2A–F. Just as for human, ionocyte clusters in each murine organ could be identified based on the highest expression of *Foxi1* (Fig. 5A–F). Observing the top 20 markers present in each ionocyte population, it was readily apparent that many of these overlapped (e.g., *Tfcp2l1*, *Hepacam2*, and *Cd24a*) (Fig. 5A–F). To further explore the overlapping genes in ionocytes derived from these six different organs, we created a matrix layout showing all intersecting DEGs (Fig. 5G). Remarkably, we found that murine ionocytes from kidney, epididymis, inner ear, prostate, airway, and salivary gland share the differential (positive) expression of 113 genes. These markers included many membrane ATPases (e.g., *Atp5h*, *Atp6ap1*, and *Atp6v1e1*) (Fig. 5H), and mitochondrial genes (e.g.,* Acss1*, *Cox8a*, and *Mdh1*) (Fig. 5I); transcription factors including those found in the human data analysis (i.e.,* Foxi1*, *Ppargc1a*, and *Tfcp2l1*); a variety of surface molecules (e.g.,* Cldn7*, *Itpr2*, and *Kit*) and signal transduction-related markers (e.g.,* Met*, *Ralgapa2*, and *Stap1*); and other markers such as dicarbonyl and l-xylulose reductase (*Dcxr*) and PDZ and LIM domain 3 (*Pdlim3*) (Fig. 5J). The full list of overlapping genes between each murine ionocyte population can be found in Supplementary File 2.

Using the DEGs computed per ionocyte population, we also evaluated the most enriched biological processes and found a clear convergence between ionocytes from the six organs studied. The most prominent processes were those involved in ion transmembrane transport, which we similarly observed using human data (Supplementary Fig. 2G).

Finally, when assessing predicted signaling from and to murine ionocytes, we observed that the main pathways signaled by ionocyte populations included amyloid precursor protein (APP), MIF, vascular endothelial growth factor (VEGF), and SEMA3 signaling, which were also found relevant in human ionocytes. As for incoming signaling, we found that KIT, PTN, FN1, and MK signaling remains relevant in both species, among others (Supplementary Fig. 3).

## Discussion

Most individual members of the ionocyte cell type ‘family’ described in this study have only recently been discovered. Consequently, there is still limited understanding of their transcriptional profile and biological function. Last year, by comparing the transcriptome of human epithelial cells in nasal, bronchial, and epididymal samples, Paranjapye et al. described conservation of ionocytes between these tissues^44^. However, a complementing cross comparison of these cell types between other organs has not previously been given. In this study, we report the presence of cells related to airway ionocytes across different human (i.e., kidneys, airways, epididymis, thymus, and skin) and murine (i.e., kidneys, airways, epididymis, inner ear, salivary gland, and prostate) organs using publicly available datasets. By comparing the transcriptomic profile of ionocytes from different organs, we observed a considerable overlap of specific DEGs (30 markers in human ionocytes and 113 in murine ionocytes). It should be noted, however, that the lists of genes characteristic of ionocytes might not be restricted to the ones found in this study. Considering technical matters, markers might have been omitted or underrated in the analysis due to several factors, including different sequencing depths between datasets, a different barcoding procedure, or the predefined p-value cutoff for DEG computation. This means that some markers, albeit not sufficiently differentially expressed, could still be relevant ionocyte signature markers. The presence of exclusive markers in the respective ionocyte populations might be the consequence of different environmental niche conditions that each population is exposed to (e.g., oxygenation, flow, or neighboring cells).

By further exploring the DEGs found in both human and murine ionocytes, we found that the main biological process enriched in ionocytes is that of ion transmembrane transporter activity, which is in accordance with their function of regulating intracellular and extracellular pH and osmolarity in aquatic vertebrate species^45–47^. Since ion transmembrane transport and regulation of acid–base homeostasis are crucial throughout the body, the presence of ionocytes is likely not limited to the organs included in our analysis. Last year, our group and others identified a population of CFTR*high*-expressing cells in the human duodenum. This rare cell population is characterized by high expression levels of *BEST4*, *GUCA2A*, and *OTOP2*, among others^48,49^. However, after identifying core ionocyte markers in the current study, we believe that these cells do not belong to the family of ionocytes as they do not express any other characteristic marker. According to the Human Protein Atlas, breast glandular cells and some cells in the thyroid express *FOXI1*^50^. Future studies should investigate whether the cells express more markers identified here.

Finally, we computed the most dominant incoming and outgoing signaling pathways in ionocytes, which were largely congruent between humans and mice (e.g., APP, SEMA3 and MK signaling), confirming a high transcriptomic conservation. Exploring ionocyte transcriptional characterization in both humans and mice enabled us to unravel the core ionocyte signature conserved in both species. Such signature included the transcription factors *FOXI1*, *PPARGC1A*, and *TFCP2L1*; the membrane ATPases *ATP6V0B*, *ATP6V1A*, *ATP6V1B1*, and *ATP6V1H*; and the surface molecules encoded by *ITPR2*, *KIT*, *KRT7*, and *ST14.*

In a recent study, Morris proposed that cell identity can be defined using three pillars: phenotype, lineage, and state^51^. Based on phenotype, which includes transcriptomic profile and functionality, it could be defended that the ionocytes identified in different organs share the same cell identity. However, the diverse developmental origins of this cell type family are an interesting observation: the kidneys and epididymis are derived from the mesodermal lineage^52,53^; the airways, thymus, and prostate are endoderm-derived^54^; and the epithelial cells of the inner ear^55^, sweat glands of the skin^56^, and the major salivary glands^57^ arise from the ectoderm germ layer. This could indicate that a convergent development has taken place, in which distinct progenitor populations give rise to the same (or a very similar) cell type.

In summary, we have identified a core signature for ionocytes, an apparent key cell type in regulating fluid ion homeostasis and pH, throughout the body. This finding can provide a better understanding or even facilitate the discovery of multi-organ disease states. One example of such an ionocyte-related disease is that caused by mutations in *ATP6V1B1*, in which patients present with severe renal tubular acidosis often accompanied by hearing loss^58,59^. A similar phenotype can be observed in patients with mutations in *ATP6V0A4*^17,60^, another core ionocyte marker, and non-surprisingly, in patients carrying *FOXI1* mutations^61^. With the recognition of ionocytes in multiple mammalian organs, additional symptoms in patients with ionocyte-related diseases might be found in previously unsuspected organs.

## Supplementary Information

Supplementary Legends.

Supplementary Information 2.

Supplementary Information 3.

Supplementary Figures.

## Data Availability

The datasets used in this study can be found in Table 1, and can be accessed either through Gene Expression Omnibus (GEO) or Synapse.
