# Author Correction: Transcriptomic profile comparison reveals conservation of ionocytes across multiple organs

**DOI:** 10.1038/s41598-023-36758-1

**Published:** 2023-06-21

**Authors:** Carla Pou Casellas, Cayetano Pleguezuelos-Manzano, Maarten B. Rookmaaker, Marianne C. Verhaar, Hans Clevers

**Affiliations:** 1 Oncode Institute, Hubrecht Institute, Royal Dutch Academy of Science (KNAW) and University Medical Center Utrecht (UMCU), Utrecht, The Netherlands; 2 Department of Nephrology and Hypertension, University Medical Center Utrecht (UMCU), Utrecht, The Netherlands; 3 Present Address: Roche Pharmaceutical Research and Early Development, 4058 Basel, Switzerland

Correction to: *Scientific Reports* 10.1038/s41598-023-30603-1, published online 02 March 2023

The original version of this Article contained an error in the Discussion section. Additionally, the Reference, which is listed below as Reference 44, was omitted. As a result,

“Most importantly, a cross-comparison of these cell types between organs has not previously been given.”

now reads:

“Last year, by comparing the transcriptome of human epithelial cells in nasal, bronchial, and epididymal samples, Paranjapye *et al*. described conservation of ionocytes between these tissues^44^. However, a complementing cross-comparison of these cell types between other organs has not previously been given.”

Reference:

44. Paranjapye, A. *et al*. Cell function and identity revealed by comparative scRNA-seq analysis in human nasal, bronchial and epididymis epithelia. *Eur. J. Cell Biol*. **101**(3), 151231. 10.1016/j.ejcb.2022.151231 (2022).

The original Article has been corrected.

