# Supplementary material for: Transcriptomic profile comparison reveals conservation of ionocytes across multiple organs
Source: Sci Rep. 2023 Mar 2;13:3516. doi: 10.1038/s41598-023-30603-1 (PMC9981729; doi:10.1038/s41598-023-30603-1)
Supplement: Supplementary file 1 — Supplementary Legends. [file 41598_2023_30603_MOESM1_ESM.docx]

**Transcriptomic Profile Comparison Reveals Conservation of Ionocytes Across Multiple Organs**

Carla Pou Casellas^1, 2^, Cayetano Pleguezuelos-Manzano^1^, Maarten B. Rookmaaker^2^, Marianne C. Verhaar^2^, Hans Clevers^1,3^*

^1^Oncode Institute, Hubrecht Institute, Royal Dutch Academy of Science (KNAW) and University Medical Center Utrecht (UMCU), The Netherlands

^2^Department of Nephrology and Hypertension, University Medical Center Utrecht (UMCU), Utrecht, The Netherlands

^3^Current address: Roche Pharmaceutical Research and Early Development, Basel 4058, Switzerland.

*Corresponding author; e-mail address: h.clevers@hubrecht.eu

**Supplementary Figure Legends**

**Figure S1. Human datasets clustering.** (A-E) Uniform Manifold Approximation and Projection (UMAP) plots depicting the clusters identified from all human datasets of either kidney, airway, epididymis, thymus, or skin tissue. (F) Clusters computed for the integrated human data (*i.e.,* all tissue datasets combined. (G) Heatmap showing the top 3 differentially expressed genes per cluster, along with the cluster identification, and the cellular composition of each cluster (orange: kidney-derived, red: airway-derived, green: epididymis-derived, purple: thymus-derived, pink: skin-derived).

**Figure S2. Murine datasets clustering and biological process enrichment in murine ionocytes.** (A-F) Uniform Manifold Approximation and Projection (UMAP) plots illustrating the cells obtained from each dataset, together with the computed clustering for murine kidney, airway, epididymis, inner ear, salivary gland, and prostate. (G) Enrichment map with the most highly enriched biological processes based on the differentially expressed genes in each murine ionocyte population.

**Figure S3. Outgoing and incoming signaling in murine ionocytes.** Heatmap depicting the main outgoing and incoming signaling pathways in murine ionocytes. Orange: kidney-derived, red: airway-derived, green: epididymis-derived, light blue: inner ear-derived, dark blue: salivary gland derived, yellow: prostate-derived.
