# Supplementary figures and images for: Transcriptomic profile comparison reveals conservation of ionocytes across multiple organs
Source: Sci Rep. 2023 Mar 2;13:3516. doi: 10.1038/s41598-023-30603-1 (PMC9981729; doi:10.1038/s41598-023-30603-1)

Supplementary Figure 1

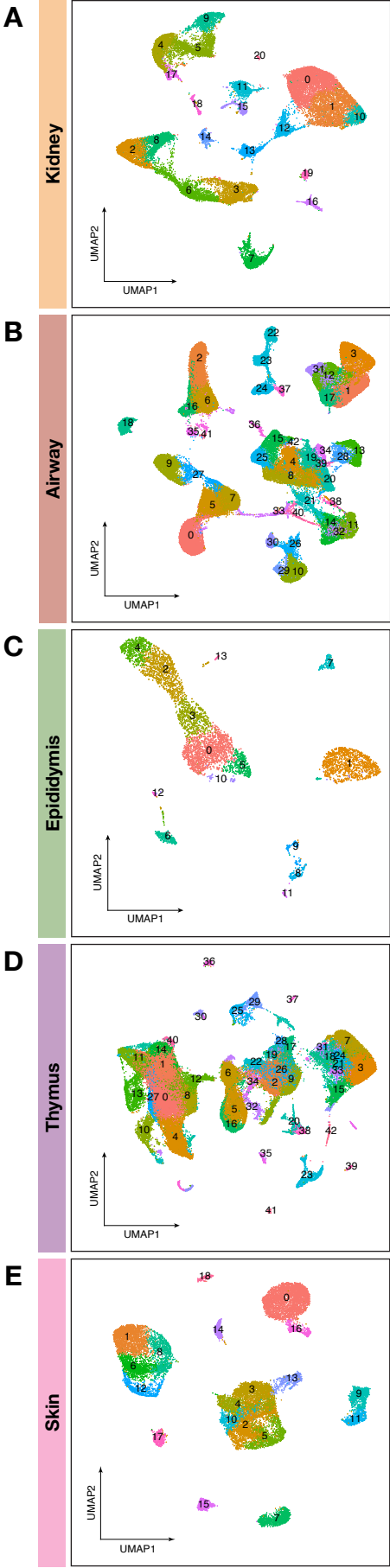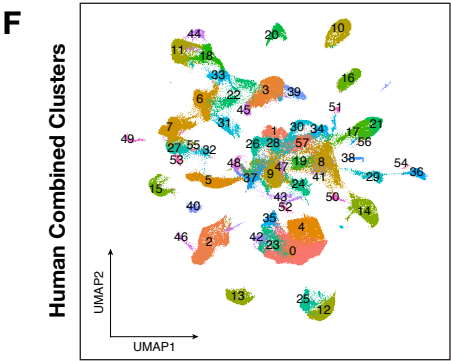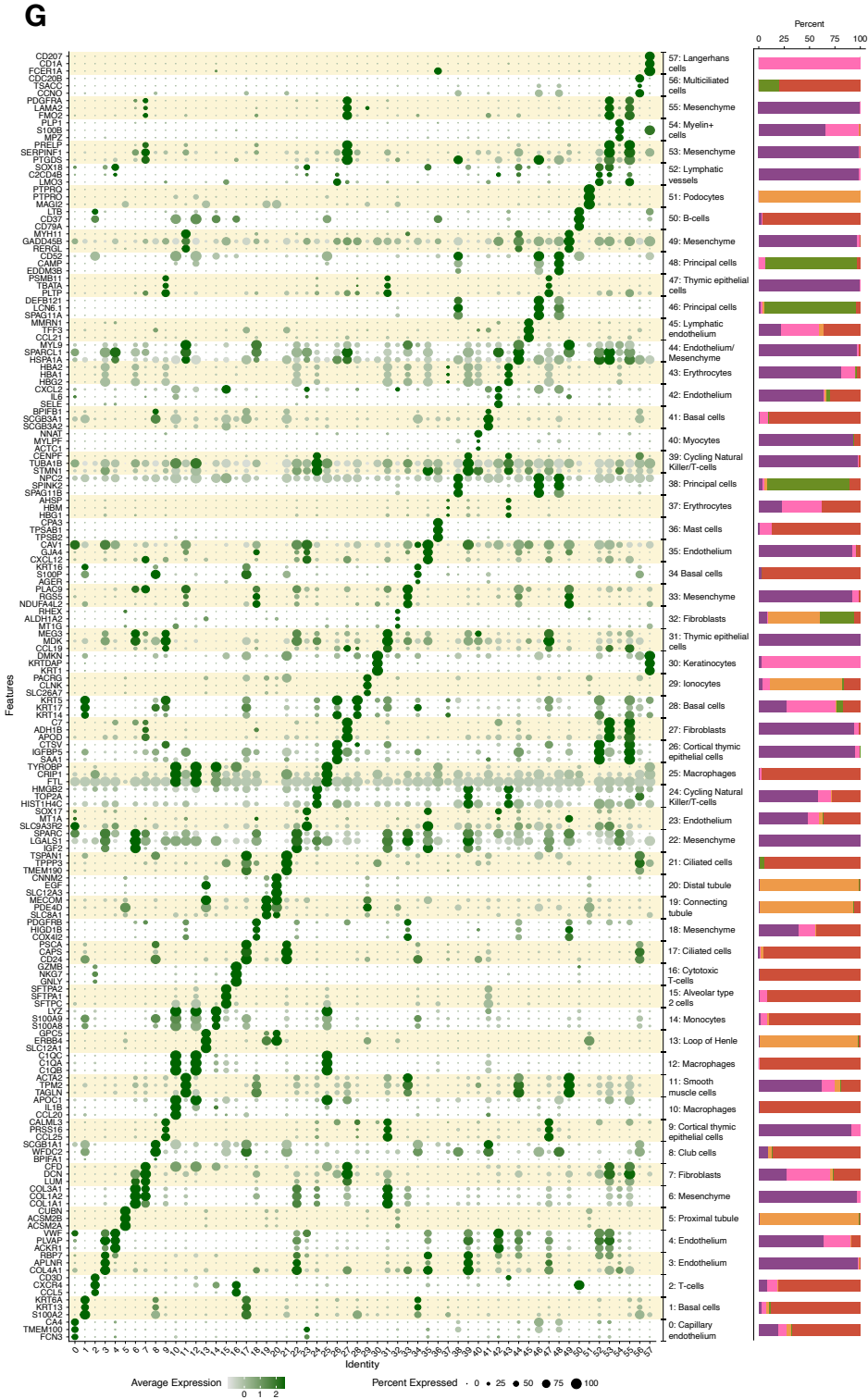

Supplementary Figure 2

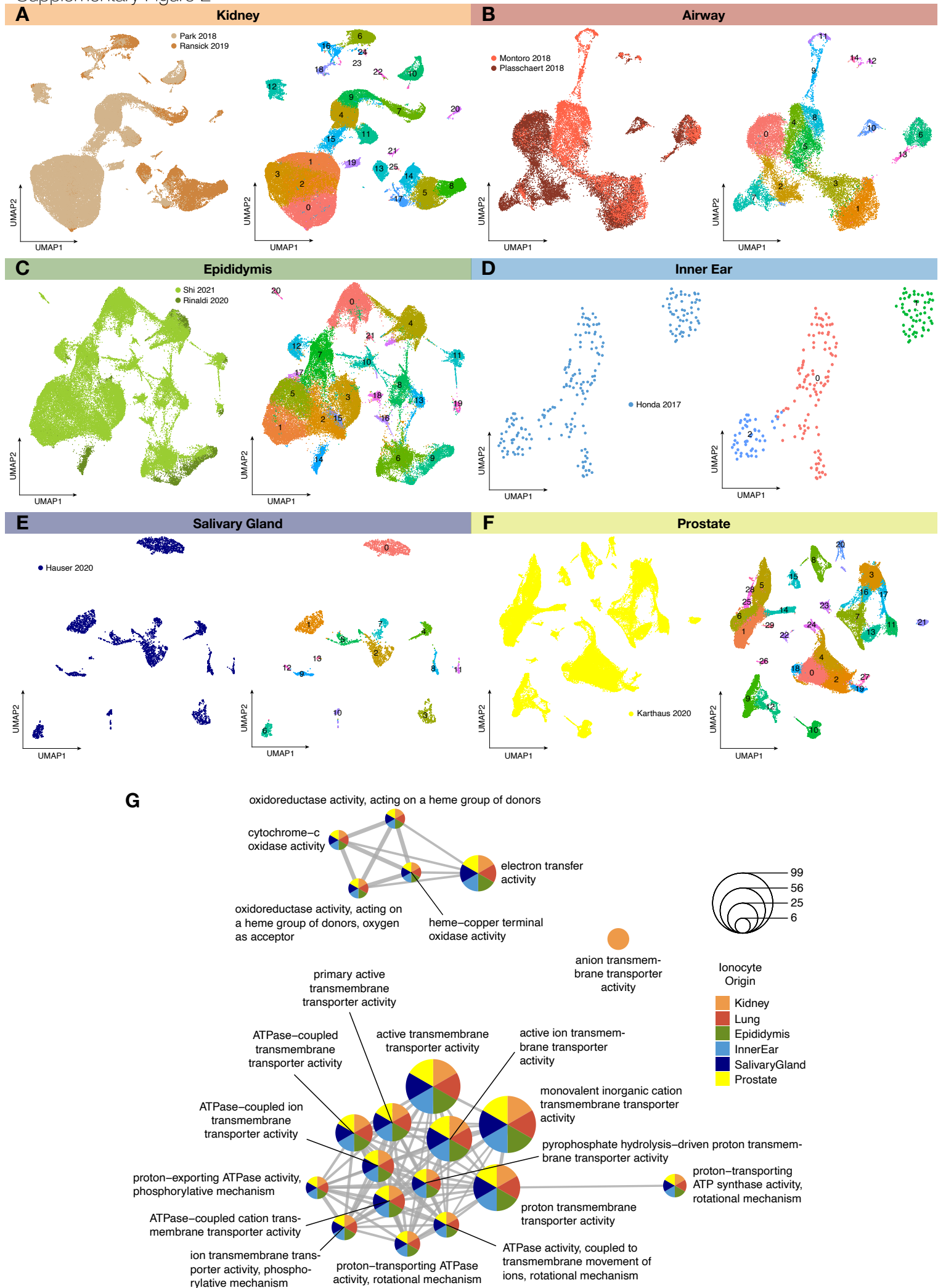

Supplementary Figure 3

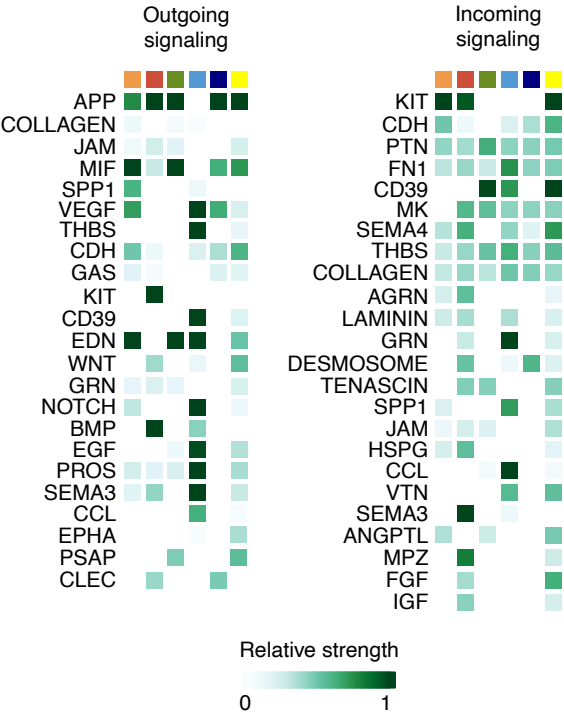

Supplement: Supplementary file 4 — Supplementary Figures. [file 41598_2023_30603_MOESM4_ESM.pdf]
